# Energy Efficient Management of Pipelines in Buildings Using Linear Wireless Sensor Networks

**DOI:** 10.3390/s18082618

**Published:** 2018-08-10

**Authors:** Sudeep Varshney, Chiranjeev Kumar, Abhishek Swaroop, Ashish Khanna, Deepak Gupta, Joel J. P. C. Rodrigues, Plácido R. Pinheiro, Victor Hugo C. de Albuquerque

**Affiliations:** 1 Indian Institute Technology, Dhanbad, Jharkhand 826004, India; sudeep149@gmail.com (S.V.); k_chiranjeev@yahoo.co.uk (C.K.); 2 Bhagwan Parshuram Institute of Technology, New Delhi 110089, India; asa1971@gmail.com; 3 Maharaja Agrasen Institute of Technology, GGSIP University, New Delhi 110078, India; ashishkhanna@mait.ac.in (A.K.); deepakgupta@mait.ac.in (D.G.); 4 National Institute of Telecommunications (Inatel), Santa Rita do Sapucaí-MG 37540-000, Brazil; 5 Instituto de Telecomunicações, 1049-001 Lisboa, Portugal; 6 Institute of Photonics and Optoinformatics, ITMO University, 197101 Saint Petersburg, Russia; 7 Graduate Program in Applied Informatics, University of Fortaleza, Fortaleza, Ceará 60811-905, Brazil; placido@unifor.br (P.R.P.), victor.albuquerque@unifor.br (V.H.C.d.A.)

**Keywords:** meta-heuristic technique, node placement, routing, wireless sensor networks

## Abstract

The efficient and safe management of air conditioner (AC), Piped Natural Gas (PNG) and water pipelines in large buildings is a major challenge for the safety of these buildings. In recent years, Linear Wireless Sensor Networks (LWSN) are being used extensively for monitoring of long natural gas, water, and oil pipelines. LWSNs can also be used for efficient and safe management of AC, PNG and water pipelines in large buildings. In this paper, a scheme for optimal placement of sensors and base stations in a linear fashion to monitor the various pipelines used in large buildings has been proposed. The proposed scheme utilizes the Lion Optimization Algorithm (LOA) and has been compared with three strategies, namely Ant Colony Optimization (ACO), Genetic Algorithm (GA) and Greedy Approach with respect to throughput, lifetime and end-to-end delay. The simulation results show that the proposed scheme exhibits better performance in comparison to the other three considered techniques for all the three parameters. The most striking feature of the proposed approach is that optimization is more effective when the length of the pipeline is more as far as end-to-end delay is concerned. The lifetime of the network is significantly improved using the proposed approach, especially when the length of the pipeline is of medium size, which makes the proposed scheme suitable for energy efficient buildings.

## 1. Introduction

In recent years, the reduction in the cost of sensor nodes has opened various new areas where Wireless Sensor Networks (WSNs) can be used. Some of the applications of WSNs include environmental, AC/oil/gas/water pipeline, river, border and health monitoring [1]. Underwater sensor networks [2,3] and software-defined wireless sensor networks [4] are also creating interest among the researchers. The nodes in WSNs have a number of constraints, e.g., limited power capability, limited communication range and small size [1]. Due to these constraints, the algorithms and techniques developed for other systems cannot be directly used without sacrificing the efficiency of the system.

In certain applications, a one-dimensional linear wireless sensor network must be established. The example of such applications includes border, oil/gas pipeline and sea/river shore monitoring. For such applications, a new subclass of sensor networks, named Linear Wireless Sensor Networks (LWSNs) has been coined by the researchers [5]. The monitoring of water, gas and oil pipelines using Linear Wireless Sensor Networks has been discussed by several researchers [6,7,8,9,10,11,12,13].

Water, gas and oil pipelines cross states, countries and sometimes also continents and their length varies between a few kilometers to thousands of kilometers. Modern large smart buildings also use various types of pipelines such as AC, water and Piped Natural Gas (PNG) pipelines, which may cross each other. The length of such pipelines varies from a few hundred meters to a few kilometers. Moreover, the network of pipelines in a smart building is a combination of various pipelines, which cross each other at different points. The number of skyscrapers is bound to increase due to the scarcity of premium space and increasing populations. Moreover, buildings are getting smarter day by day. The reduced cost of sensors has made it possible to extensively use sensors for monitoring and safety purposes in smart buildings. Recently, Kumar et al. [14] published a survey to describe the techniques currently being used to improve the performance of sensor-based systems used for applications in intelligent buildings such as improving air quality, lighting, heating/cooling, ventilation, power management, water management, and cooking gas management.

In LWSNs, the nodes are placed in a strictly linear or semi-linear form. Due to their special topology and application requirements, solutions used for ordinary sensor networks cannot be directly applied to LWSNs. The LWSNs can be classified either based upon topology (thick/thin/very thin) or based upon hierarchy (one level/two level/three level) [15].

Once an LWSN is deployed, it may not be feasible or cost effective to change the power source in sensor nodes frequently. Thus, while deploying the LWSN one of the most critical and challenging tasks is to deploy sensor nodes effectively so that the deployed network must be long-lived with minimum energy consumption. The major research challenges in LWSNs include node placement, coverage, topology, routing, lifetime optimization, and energy efficiency [5]. Some of the issues are inter-related with each other, for example efficient node placement will increase the coverage and lifetime of the network. Similarly, an efficient routing protocol will save energy and hence the lifetime of the network.

Node placement, routing, and lifetime optimization are important research problems in the LWSN field. In [16,17,18,19,20,21,22,23] various node placement schemes and routing protocols for increasing the lifetime of LWSNs have been proposed. The optimal node placement plays an important role in the energy efficiency and lifetime of a network. Therefore, in recent years several optimization methods have been applied for this purpose.

In [24], Guo et al. presented a greedy approach-based technique for optimal sensor placement in an oil pipeline. Their main objective was to maximize the lifetime of the linear sensor network. The authors used a simple equal distance placement scheme and equal power placement scheme and proposed two placement heuristics-based algorithms using a greedy approach.

Meta-heuristic optimization techniques are turning out to be useful in designing applications using LWSNs. Some of the major meta-heuristic techniques are single solution-based meta-heuristics and population-based meta-heuristics [25]. Nature-inspired techniques are an important subclass of population-based meta-heuristics. Therefore, several researchers have used nature-inspired techniques in various applications. Recently, Kaur-Mahajan [26] used hybrid meta-heuristic techniques to design energy efficient protocols for WSNs.

Rabei et al. [27], used nature-inspired techniques for node placement and lifetime optimization of a Linear Wireless Sensor Network specifically used in monitoring of oil pipelines. The problem of the optimal number of sensors to be deployed with a given initial energy of each sensor node and message buffering limitations has been discussed. The author used two evolutionary algorithms to solve the deployment problem, i.e., Genetic Algorithms (GA) and Ant Colony Optimization (ACO).

The solutions proposed for very long oil/gas pipelines crossing states, countries, and continents cannot be directly applied effectively for managing pipelines inside a building. Moreover, the greedy approach is heuristic in nature, and ACO follows a strictly linear path, which does not consider the crossing nature of pipelines inside a building.

In [28], Yazdani-Jolai presented the Lion Optimization Algorithm to be used for optimization problems. Considering the nature of pipelines inside a large building, LOA seems to be a good approach for optimizing nodes placement. Therefore, in this paper, an efficient node placement strategy for effective and efficient pipeline management inside a large smart building using LOA has been proposed. The major contributions of the paper are as follows:

- The paper proposes a solution to manage various pipelines inside a large building effectively.
- Lion Optimization Algorithm (LOA) has been used to optimize the node placement. To the best of our knowledge, LOA has not been used in the literature for efficient node placement for LWSN.
- An efficient mechanism for data transfer for such pipeline has been designed.
- The performance comparison of the proposed scheme with three techniques namely greedy approach, ACO and GA [27] with respect to lifetime, throughput and end-to-end delay has been performed.

**Figure 1:**
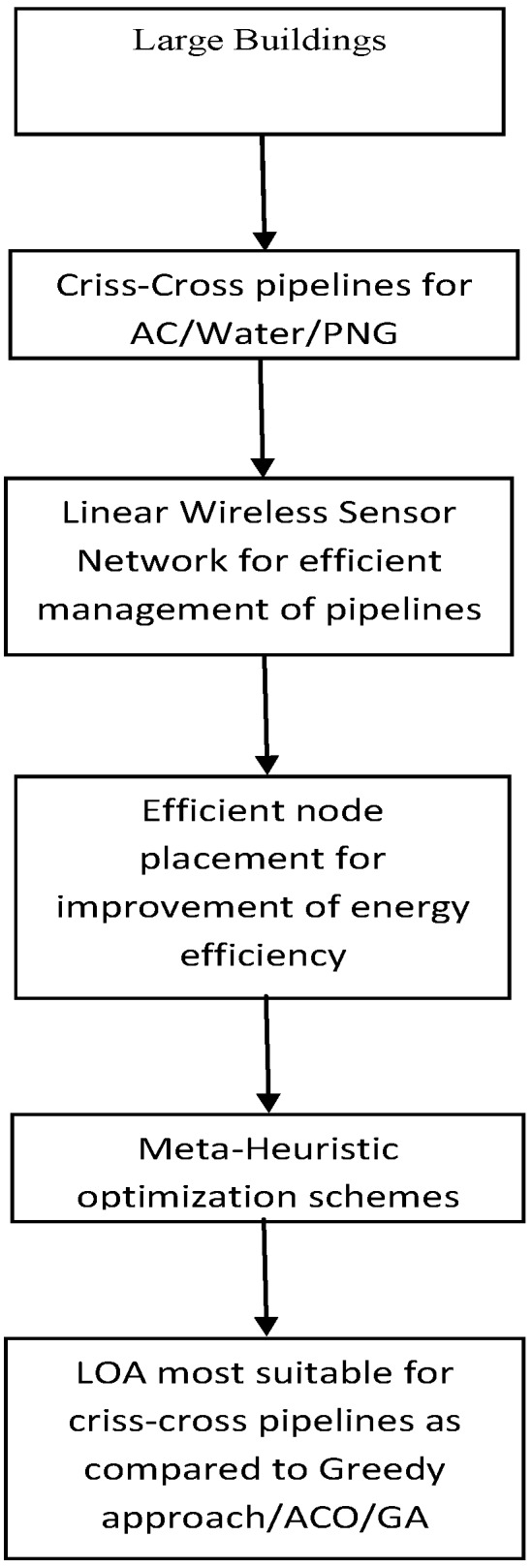
Proposed Problem and Solution.

Figure 1 explains the path to arrive at the proposed approach. The rest of the paper is organized as follows: Section 2 presents the problem statement whereas proposed solution and simulation/ result analysis are given in Section 3 and Section 4, respectively. Section 5 presents the conclusions of the paper and points out possible future work directions.

## 2. Problem Statement

**Figure 2:**
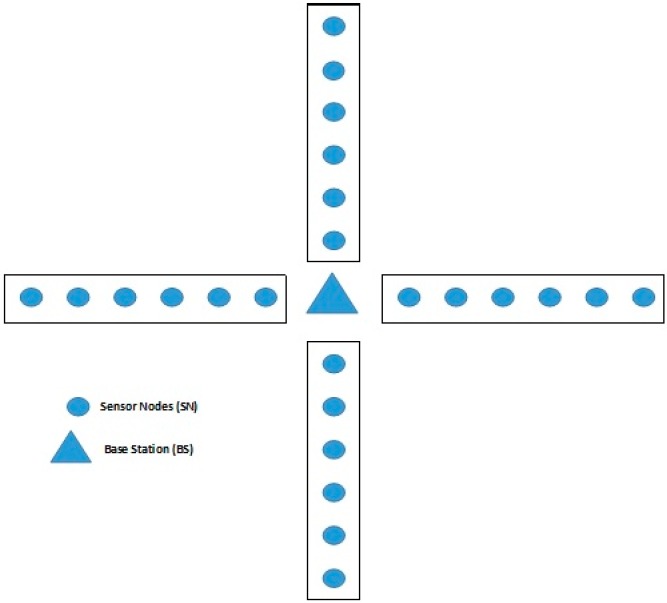
Multiple straight pipelines.

We consider a straight section of pipeline (AC/water/gas/oil) which is enclosed with two stations, one at each end of the section of the pipeline. The pipeline (channel) can run for a few meters to a few kilometers. This channel should be effectively checked; at the end of the day, the entire length of the channel should be secured, and the detected data should be transmitted to a specific node named sink or base station. The sink node should be at one of the stations. In this manner, it is accepted that there is a pipeline with length L, with sensor nodes SN (N1, N2... Nn) and two stations are disseminated at the closures of the channel. A sink node S should be placed at one of the channel′s closures near one of the stations. Sensors and also the sink node are expected to have restricted abilities including the communication range and the size of the buffer. Here, two scenarios exist, one is to establish a new pipeline to connect two stations efficiently, and the other is to efficiently manage the connection between existing stations. The problem can be generalized in that there are multiple channels, which are interconnected with each other in some pre-specified manner. This paper discusses the establishment of a network of pipelines or channels with minimum number of nodes such that the entire area is covered in a secure, robust manner and data is transferred to the BS with a minimum number of nodes. The energy efficiency and lifetime of the network are also considered while designing the solution of the proposed problem. For setting up a pipeline, there are multiple straight pipelines connected in a manner to common BS as shown in Figure 2.

## 3. Proposed Solution

In recent years, researchers have used evolutionary approaches such as Genetic Algorithm and Ant Colony Optimization (ACO) [27] for solving the deployment problem in LWSNs. In [27], the authors claimed that evolutionary algorithms can solve the node deployment problem in LWSNs effectively and efficiently. Recently, a new evolutionary approach namely Lion Optimization Algorithm [28] has been proposed which has been proven better than the other existing techniques and has been used in several areas. The Ant Colony Optimization is more suitable for a single straight pipeline because the ants move in a straight-line one behind the other in one direction only. However, the problem considered in the paper includes multiple pipelines, and the data is to be sent to the Base Station (BS) from multiple directions by the sensor nodes as shown in Figure 2. The group hunters such as lions attack the prey from multiple directions, and the Lion Optimization Algorithm tries to use this characteristic of lions to find the optimal solution of the problem being considered. This motivated us to use Lion Optimization Algorithm (LOA) for optimal node placement and Base Station (BS) in LWSN including multiple pipelines. To the best of our knowledge, LOA has not been used by any other researchers for this purpose.

### 3.1. Lion Optimization Algorithm

The Lion Optimization Algorithm, first described by Yazdani and Jolai [28] is a population-based meta-heuristic calculation. This calculation is based on the chasing and social conduct of the lion. Lions are arranged in two sorts of social associations, one is the pride and other is wanderers. The prides are gatherings of lions while the wanderers move sporadically either in pairs or independently.

The behavior of a wanderer that moves independently describes the exploration search while the pride behavior represents the exploitation search. The use of both identifies a balance between the exploitation and exploration search, which suggest better convergence towards global optima.

In LOA, every arrangement is set apart as a lion given as $=\;\left[s_{1},s_{2},s_{3},\ldots \ldots \ldots s_{n}\right]$. The cost capacity C is utilized to assess the expense of every lion. At first sensor node (SN) arrangements are produced haphazardly in pursuit space in which %*P* are the wanderers and rest are separated into c bunches of prides. The %f in every pride is females and the remainder are the males. The tertiary of the pride is characterized by the best gone-by position of every lion [28].

The chasing style of the pride is particular to trap the quarry. A couple of females from the pride assault the quarry while others move from region to region. The chasing individuals from the pride partition themselves into three gatherings, i.e., focus, left and right area. The gathering utilizes the resistance-based learning [29] to assault the quarry. The gathering with most extreme wellness worth is considered as the middle gathering and position of the quarry is identified with the position of chaser as appears in Equation (1):

(1) Pquarry=∑pchasers(s1,s2,s3,………snv)number of chasers

Here, $P_{quarry}\;\mathrm{and}\;p_{chaser}$ are the position of the quarry and chaser respectively. During the hunting process, the chaser are elected randomly and position updating of the quarry and chaser (left, right and center section) is given by Equation (2) (where percentage *_i_* is the percentage of improvement in fitness of chaser), (3) and (4) respectively:

(2) $$p_{quarry}{}^{\prime }=p_{quarry}+rand*precentage_{i}*\left(p_{quarry}-P_{chaser}\right)$$

(3) $$\begin{array}{l} P_{chaser}{}^{\prime }\\ =\{\begin{array}{l} rand*\left(2*p_{quarry}-P_{chaser}\right)+p_{quarry}\;if\;\left(2*p_{quarry}-P_{chaser}\right)<p_{quarry}\\ rand*p_{quarry}+\left(2*p_{quarry}-P_{chaser}\right)\;if\;\left(2*p_{quarry}-P_{chaser}\right)>p_{quarry} \end{array} \end{array}$$

(4) $$P_{chaser}{}^{\prime }=\{\begin{array}{c} rand*P_{chaser}+p_{quarry}\;if\;P_{chaser}<p_{quarry}\\ rand*p_{quarry}+\;P_{chaser}\;if\;P_{chaser}>p_{quarry} \end{array}$$

The random function (rand) is used to generate a number between 0 and 1. Equation (3) is used to update the position of the chaser if it is in right or left section otherwise Equation (4) is used for the same. The remaining females (which are moving towards territory) update their position according to Equation (5):

(5) $$P_{female\_lion}{}^{\prime }=P_{female\_lion}+2*distance*\vec{R1}+U\left(-1,1\right)*\tan\left(\theta \right)*distance*\vec{R2}$$

Here, $P_{female\_lion}{}^{\prime },P_{female\_lion}$ is the updated and original position of the female lion respectively. The distance is the distance between the $P_{female\_lion}{}^{\prime }$ and the point selected by tournament strategy. $\vec{R1}$ is the vector representing the start point, $\vec{R2}$ is selected such that $\vec{R1}.\;\vec{R2}=0$ and $\|\vec{R2}\|=1$. Theta (θ) varies uniformly between −π/6 to π/6 radian to widen the area of current solution. U(−1,1) is used for the generate −1 or 1 depending upon the direction of the resident male roaming in same or opposite direction of the pride territory respectively.

The tournament process enables the pride to change its size in each iteration by using Equation (6):

(6) Sizepi=max(2,ceil(inoli2)) i = 1, 2, 3……N

where, $inol_{i}$ is the number of lions in pride *i* with improved fitness in previous iteration is given by Equation (7):

(7) $$inol_{i}=\sum_{j=1}^{n}sucess\left(j,iter,N\right)\;i\;=\;1,\;2,\;3\ldots \ldots N$$

Here the sucess(j,iter,N) gives the success of lion *j* in group *N* at iteration, iter denoted by (8):

(8) $$sucess\left(j,iter,N\right)=\{\begin{array}{l} 1\;best_{j,N}^{iter}<best_{j,N}^{iter-1}\\ 0\;best_{j,N}^{iter}=best_{j,N}^{iter-1} \end{array}$$

The nomad lion moves randomly in the search space (explorative search) used to avoid local optima. The movement of the *i*th nomad in the *j*th is represented by Equation (9):

(9) $$nomad_{ij}^{lion}=\{\begin{array}{c} nomad_{ij}^{lion}\;if\;rand>pr_{i}\\ rand\;else \end{array}$$

Here, the $pr_{i}$ is the probability generated for *i*th nomad given by (10):

(10) $$pr_{i}=0.1+min\left(0.5,\frac{nomad_{i}-Best_{nomad}}{Best_{nomad}}\right)\;i\;=\;1,\;2,\;3\ldots .\;\mathrm{no}.\;\mathrm{of}\;\mathrm{nomads}$$

where $nomad_{i}$, $Best_{nomad}$ is the cost of current position of *i*th nomad and best nomad, respectively.

The way of life exchanging of the lion happens because of various reasons. The prime male in the pride may defeat another male of the same pride, or any wanderer may beat the main lion of the pride. The defeated lion leaves the pride. The male and female lions will die with time. Some male lions change their pride to take over another pride. Some females relocate from one pride to another. This may change the quantity of migrants and number of individuals in a specific pride. This concept of the LOA algorithm which can be implemented in LWSN for placing the nodes at optimized positions and transferring the data is discussed in next section.

### 3.2. Node Deployment in LWNS Using

In this section, a fitness function and an algorithm for node deployment problem in a network of pipelines using Lion Optimization Algorithm is presented.

#### 3.2.1. Fitness Function for Node Deployment

Let the length of a section of pipeline be *L*. The number of nodes to be used in any given length can be calculated as:

(11) $$Min_{No\_nodes}=\frac{L}{2*R}$$

Here, *R* is the communication range of each node. $Min_{No\_nodes}$ is the minimum number of nodes required to produce a connected network during transmission. This process needs the deployment of nodes at a distance of 2*R. However, this process needs to be optimized such that the network remains connected even in the case of node failure. This is done by using the backup nodes placed in the pipeline at a distance of 2*R. So, we have:

(12) $$No_{nodes}=\frac{L}{2*R}+\frac{L}{2*R}=\frac{L}{R}$$

Here, the $\frac{L}{2*R}$ are the backup nodes used to backup the communication. The nodes may change their roles if the reverse direction flow occurs. Nodes near the sink (leader nodes) assume the liability of sending their information and sending the majority of the received messages from different nodes. Along these lines, the sent sensor systems lifetime relies on the vitality of the nodes near the sink. In the meantime, the sent nodes experience the ill effects of little support size where messages could be dropped because of non-accessibility of spare space, so the nodes near the sink consume large amounts of energy so the volunteer nodes are placed nearer the sink nodes. Moreover, these volunteer nodes are also required to check for sudden changes in the pressure to block the current flow and prevent losses due to leaks.

The node placement in the pipelines of the systems is optimized using the Lion Optimization Algorithm, which is a meta-heuristic technique. Here, the number of sensor nodes is considered to be the number of lions, which consists of prides as well as wanderers. The base station (BS) is considered as the prey which is to be approached by all the other lions (sensor nodes). The number of pipelines surrounds the base station. One node closest to the base station is considered to be the leader node which is directly connected with the BS. The concept of attacking by the pride comes into play when the data has to be transferred by the nodes.

The corresponding fitness to deal with the optimization of positioning the sensor nodes in LWSN is as follows:

F=α1dist+α2∗delay+α3∗drop_ratio

such that:

$$\alpha_{1}+\alpha_{2}+\alpha_{3}=1$$

*dist* is the distance of the node from the previous node; *delay* is the end-to-end delay and drop ratio is the ratio of the number of packets dropped to the total number of packets sent. This function is a minimization function. The target is to place the node the farthest with minimum *delay* and *drop ratio*. The optimization function is executed in two scenarios: one is the normal and other is after ignoring the neighbor the current node. The average value of the two-fitness function is used as the decision factor. Here, $\alpha_{1}=0.3,\;\alpha_{2}=0.3,\;\alpha_{3}=0.4$ taken is based on experiments. The whole concept can be easily understood by using the following algorithm:

#### 3.2.2. Node Deployment Algorithm

The pipeline setup can be considered as a tuple of following fields: (a) Location of base station (BS). (b) Communication range of sensor node R. (c) The starting coordinate of the channel in X direction. (d) The end coordinate of the channel in the X direction. (e) The starting coordinate of the channel in the Y direction. (f) The end coordinate of the channel in the Y direction.

Pipeline setup (BS,R, xMin, xMax, yMin, yMax)

The proposed algorithm setups a pipeline with number of sensor nodes with transmitting range R and a Base Station (BS). The algorithm decides the position of each sensor node and the BS. This pipeline is to be setup with area (*xMax* − *xMin*)*(*yMax* − *yMin*)

The systematic algorithm is presented below:

(1) Calculate number of nodes in the network
  $$n=\frac{L}{R}$$
  where:
  $$L=\sqrt{{\left(xMax-xMin\right)}^{2}+{\left(yMax-yMin\right)}^{2}}$$
(2) Initialize the Network
  P_nodes_(:,1) = rand(*n*,1)*(*xMax* − *xMin*) + *xMin*
  P_nodes_(:,2) = rand(*n*,1)*(*yMax* − *yMin*) + *yMin*
  Here, rand(*n*, 1) gives n random numbers ranging from [0,1]
(3) $P_{nodes}^{\prime }=P_{nodes}$
(4) Initialize the position of the base station
  PBS=∑pnodesn
(5) Transfer the data from nodes to BS and calculate dist, delay and drop_ratio as:
  $$dist=\sum dist_{i}$$
  $$dist_{i}=\sqrt{{\left(BS\left(1,1\right)-P_{nodes}^{\prime }\left(i,1\right)\right)}^{2}+{\left(BS\left(1,2\right)-P_{nodes}^{\prime }\left(i,2\right)\right)}^{2}}$$
  delay = Timeofpacketarrival−Timeofpacketgeneration
  dropratio=1−nos.ofPktGeneratedNos.ofPktreceivedatBS
(6) Calculate
  F=α1dist+α2∗delay+α3∗drop_ratio
(7) Transmit a one-hop hello signal from the BS then:
  no_of_leader = number of acknowledgement Received.
(8) If (no_of_leader > 1)
  - $P_{nodes}{}^{\prime }=\{\begin{array}{l} rand*\left(2*p_{BS}-P_{nodes}\right)+p_{BS}if\left(2*p_{BS}-P_{nodes}\right)<p_{BS}\\ rand*p_{BS}+\left(2*p_{BS}-P_{nodes}\right)if\left(2*p_{BS}-P_{nodes}\right)>p_{BS} \end{array}$
  - $p_{BS}{}^{\prime }=p_{BS}+rand*precentage_{i}*\left(p_{BS}-P_{nodes}\right)$
    Else:
      - $p_{i}^{node}{}^{\prime }=\{\begin{array}{c} p_{i}^{node}ifrand>pr_{i}\\ randelse \end{array}$
    Here, $pr_{i}=0.1+min\left(0.5,\frac{p_{i}-Best_{pi}}{Best_{p}}\right)$
      ii. $P_{nodes}{}^{\prime }=∐P_{i}^{node}$
        $$p_{BS}{}^{\prime }=p_{BS}+rand*precentage_{i}*\left(p_{BS}-P_{nodes}\right)$$
    end if
(9) Transfer the data from nodes to BS and calculate dist, delay and drop_ratio as:
  $$dist=\sum dist_{i}$$
  $$dist_{i}=\sqrt{{\left(BS\left(1,1\right)-P_{nodes}^{\prime }\left(i,1\right)\right)}^{2}+{\left(BS\left(1,2\right)-P_{nodes}^{\prime }\left(i,2\right)\right)}^{2}}$$
  Delay = Timeofpacketarrival−timeofpacketgeneration
  dropratio=1−nos.ofPacketGeneratedNos.ofPacketreceivedatBS
(10) Calculate:
  F′=α1dist+α2∗delay+α3∗drop_ratio
(11) If(*F*′ < *F*)
  Then:
    $$P_{BS}=p_{BS}{}^{\prime }$$
  and $P_{nodes}=P_{nodes}^{\prime }$
  end if
(12) If (|F−F’|<0.01)
  Then exit
  Else go to step 3

The above algorithm first estimates the number of nodes in the network based on the length of the pipeline. Initially, the position of nodes is generated randomly on the pipeline. After that, the Lion Optimization meta-heuristic is used to get the optimized position of the nodes. The mathematical details have been discussed in the previous subsection. While optimizing the position of nodes the drop ratio and delay are considered, i.e., the position having optimized delay and drop ratio is selected.

## 4. Performance Evaluation and Result Analysis

The algorithm described in the previous section has been implemented using MATLAB. The proposed approach has been compared with different existing techniques such as ACO, GA and Greedy Approach. The parameters considered for comparison are delay, throughput and the normalized lifetime. The length of pipeline has been varied from 500 m to 3500 m. The initial energy of each sensor node has been considered to be 1 joule, the energy consumed in communication is directly proportional to communication distance. The parameters considered are similar to the closest work [16]. The simulation experiments have been repeated for sensor node communication range varying from 25 m to 100 m.The parameters considered have been summarized in the Table 1.

**Figure 3:**
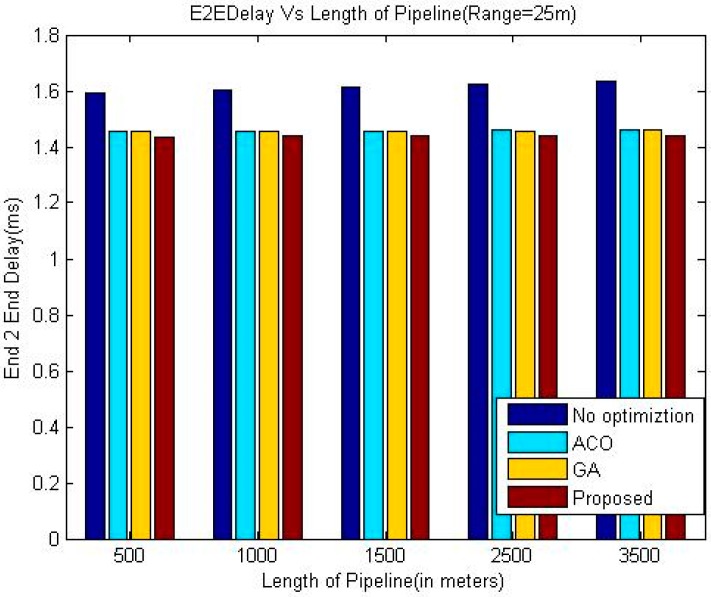
Comparison of End-to-End delay (Range 25 m).

**Figure 4:**
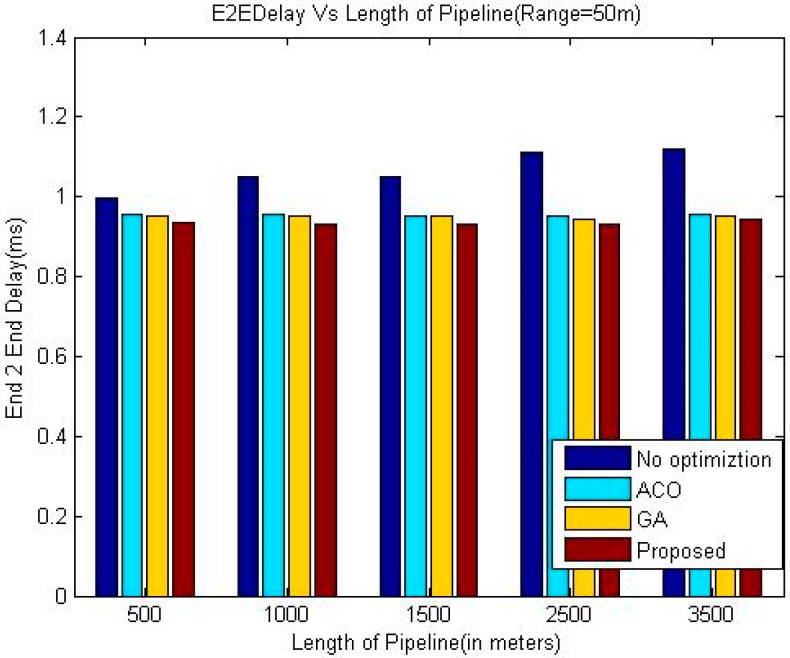
Comparison of End-to-End delay (Range 50 m).

**Figure 5:**
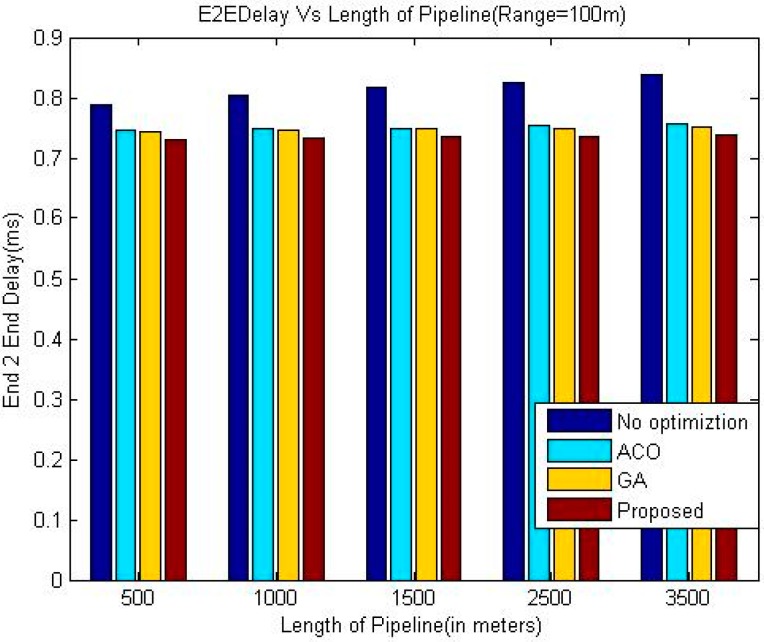
Comparison of End-to-End delay (Range100 m).

The comparison of end-to-end delay between proposed technique and other three techniques namely ACO, GA and greedy approach has been shown in the Table 2, Table 3 and Table 4 and graphically in Figure 3, Figure 4 and Figure 5.

The simulation results shown in Figure 3, Figure 4 and Figure 5 indicate that the proposed algorithm using LOA outperforms greedy approach and exhibits slightly better performance than GA and ACO as far as the delay is concerned. It is due to the dynamic setup of the pipeline by considering different factors, which is a special characteristic of LOA. As expected, the End-to-End delay is decreasing when the communication range of the sensor nodes is increases because of the reduced number of intermediate nodes required to deliver a data packet.

It is evident from the simulation results that the delay in the greedy approach is increasing with the length of the pipeline whereas this trend is not consistent in the case of the other three meta-heuristic techniques. A possible reason for this phenomenon may be that the number of nodes also increases when the length of the pipeline is increased. In the case of meta-heuristic techniques, when the number of nodes in the system is higher, the meta-heuristic techniques can be applied more efficiently which may offset the effect of increased length of the pipeline on the end to end delay in some cases.

The proposed algorithm outperforms greedy approach as far as throughput is concerned. Additionally, the throughput of our technique is slightly better than the other two considered techniques namely GA and ACO. In the presented approach, the location has been optimized based upon drop ratio and delay whereas the authors in [24] try to optimize the location based upon delay only. This factor is the reason for the better throughput of the proposed algorithm. It is evident from the simulation results that the throughput increases upon increasing the communication range of sensor nodes for all the algorithms.

**Figure 6:**
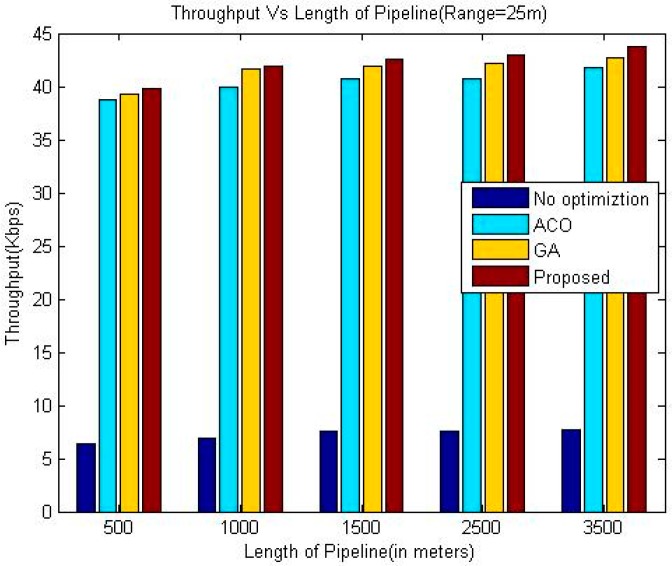
Comparison of Throughput (Range 25 m).

**Figure 7:**
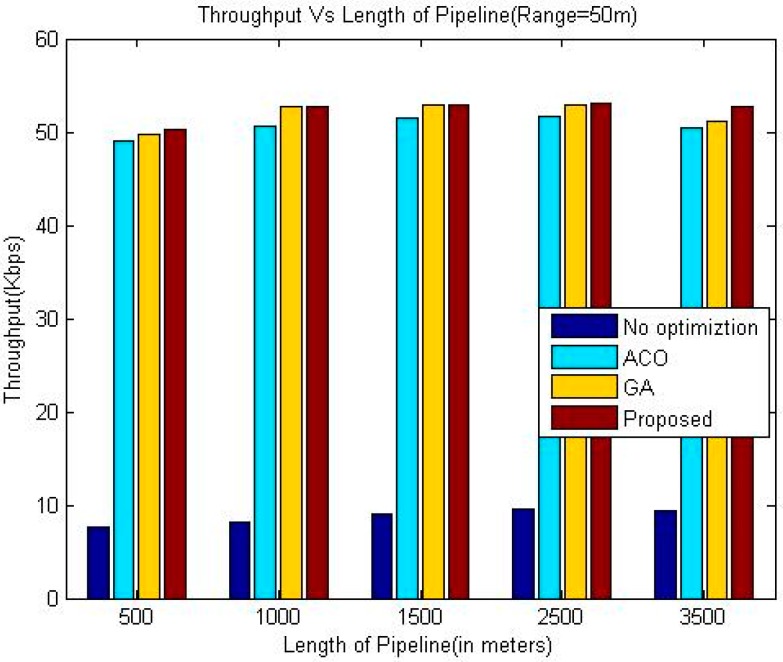
Comparison of Throughput (Range 50 m).

**Figure 8:**
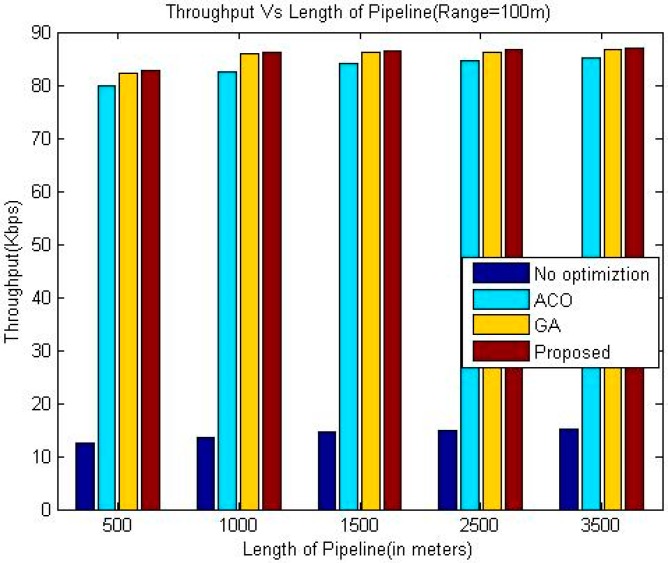
Comparison of Throughput (Range 100 m).

**Figure 9:**
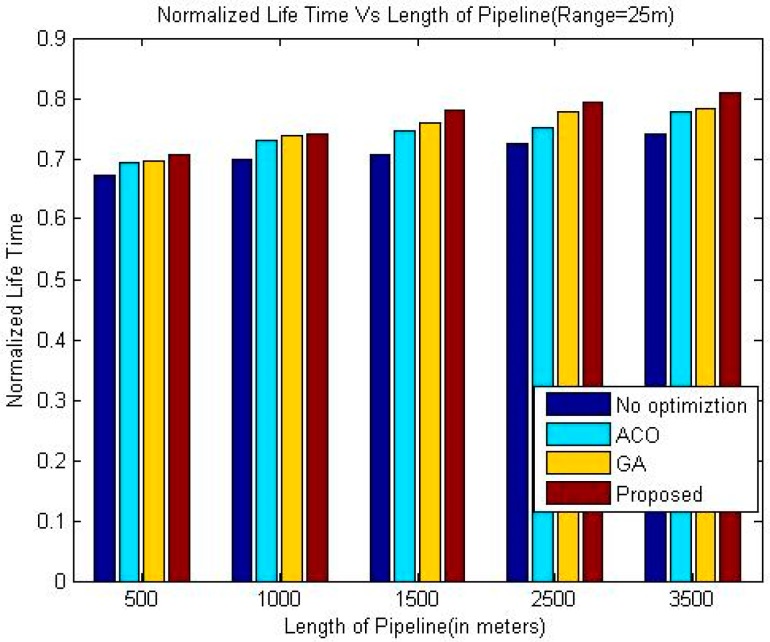
Comparison of Normalized Lifetime (Range 25 m).

**Figure 10:**
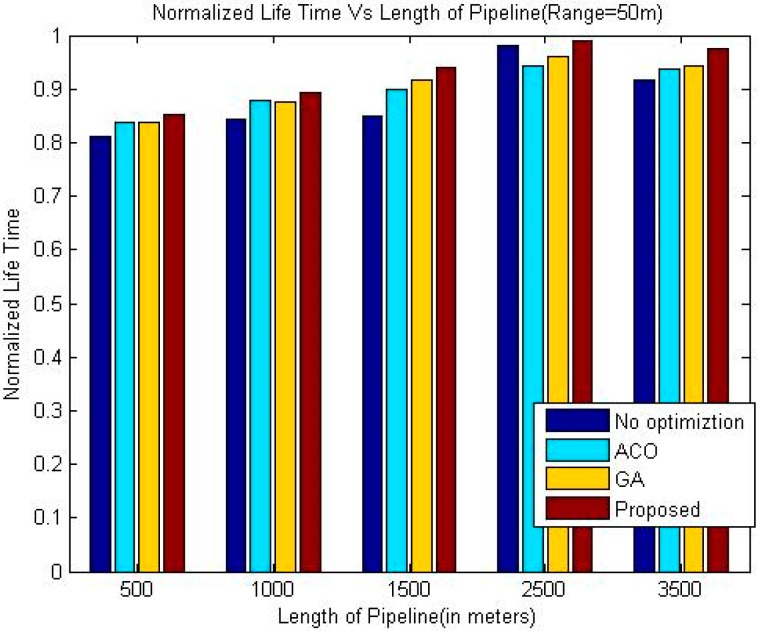
Comparison of Normalized Lifetime (Range 50 m).

**Figure 11:**
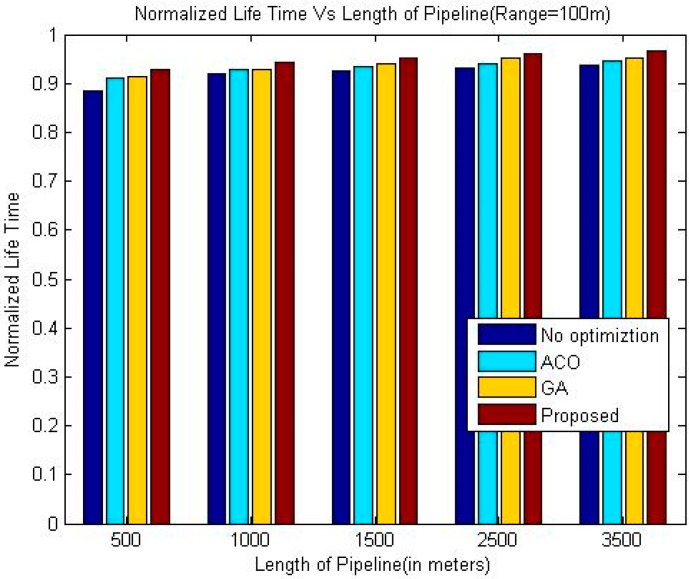
Comparison of Normalized Lifetime (Range 100 m).

The comparison of the throughput is shown in Table 5, Table 6 and Table 7. Whereas it has been shown graphically in Figure 6, Figure 7 and Figure 8. The comparison of the normalized life time is shown in Table 8, Table 9 and Table 10. The Figure 9, Figure 10 and Figure 11 represents the comparison of normalized lifetime between various algorithms graphically.

The normalized lifetime of the proposed techniques signifies that lifetime of the network in each scenario with different pipeline length is better than the other techniques under consideration. The reason behind this phenomenon is like the nature of pipelines inside a building; LOA also considers crossing paths for optimization. The performance is significantly better than all other three techniques under consideration when the length of the pipeline is 1500 m, which makes it suitable for pipelines inside a large building.

When we increase the communication range of the sensor nodes the lifetime of the network increases slightly for all the algorithms. Although, the energy consumed per data packet is proportional to the communication range of sensor nodes, however, due to reduced number of intermediate nodes, the total energy requirement to transfer a data packet is less. Therefore, the normalized lifetime shows improvement upon increasing the communication range of the sensor nodes.

## 5. Conclusions

The paper presents an algorithm to set up a network of straight pipeline segments crossing each other and efficient deployment of sensor nodes by using the Lion Optimization Algorithm. The pipeline setup using the proposed algorithm exhibits better delay, lifetime and throughput of the network as compared to the ant colony optimization and genetic algorithm for varying length of pipeline. The proposed approach is particularly suitable for the pipelines used inside a large building as clear from the simulation results. Although the proposed approach has been validated using simulation experiments, the field study of the proposed approach is left as future work. One may explore the suitability of other meta-heuristic techniques and hybrid meta-heuristic techniques for node placement in crossing pipelines inside a large building.

## Figures and Tables

**Table 1:** Simulation parameters.

Parameters	Value
Area	500–3500 * 500–3500 m^2^
Channel type	Wireless channel
Radio propagation model	Two Ray Ground
Initial Energy	1 J
Pipeline Length	500, 1000, 1500, 2500, 3500 m
Max packet in in queue	50 (packets)
Communication Range of nodes	25, 50, 100 m

**Table 2:** Comparison of Delay (Range 25 m).

Length of Pipeline (Meters)	End-to-End Delay (in msec.)			
	Greedy Approach	ACO	GA	Proposed
500	1.5913	1.4554	1.4548	1.4318
1000	1.6039	1.4560	1.4554	1.4378
1500	1.6140	1.4570	1.4563	1.4386
2500	1.6261	1.4586	1.4572	1.4398
3500	1.6334	1.4610	1.4597	1.4413

**Table 3:** Comparison of delay (Range 50 m).

Length of Pipeline (Meters)	End-to-End Delay (in msec.)			
	Greedy Approach	ACO	GA	Proposed
500	0.994935	0.956497	0.953487	0.936497
1000	1.04822	0.955523	0.953221	0.933323
1500	1.04837	0.953575	0.952514	0.931575
2500	1.11316	0.952708	0.943897	0.931086
3500	1.11988	0.955553	0.953412	0.943323

**Table 4:** Comparion of delay (Range 100 m).

Length of Pipeline (Meters)	End-to-End Delay (in msec.)			
	Greedy Approach	ACO	GA	Proposed
500	0.7870	0.7461	0.7437	0.7301
1000	0.8036	0.7483	0.7465	0.7321
1500	0.8176	0.7498	0.7479	0.7346
2500	0.8243	0.7537	0.7498	0.7362
3500	0.8385	0.7553	0.7512	0.7378

**Table 5:** Comparison of Throughput (Range 25 m).

Length of Pipeline (Meters)	Throughput (in KBps)			
	Greedy Approach	ACO	GA	Proposed
500	6.33	38.70	39.32	39.77
1000	6.85	40.00	41.68	41.90
1500	7.51	40.73	41.97	42.54
2500	7.59	40.78	42.12	42.99
3500	7.66	41.81	42.68	43.71

**Table 6:** Comparison of Throughput (Range 50 m).

Length of Pipeline (Meters)	Throughput (in KBps)			
	Greedy Approach	ACO	GA	Proposed
500	7.63	48.99	49.77	50.34
1000	8.24	50.63	52.76	52.78
1500	8.99	51.56	52.87	52.96
2500	9.61	51.62	52.94	53.03
3500	9.33	50.39	51.12	52.80

**Table 7:** Comparison of Throughput (Range 100 m).

Length of Pipeline (Meters)	Throughput (in KBps)			
	Greedy Approach	ACO	GA	Proposed
500	12.44	79.85	82.13	82.85
1000	13.43	82.53	86.00	86.13
1500	14.65	84.04	86.18	86.42
2500	14.96	84.54	86.29	86.74
3500	15.21	85.14	86.73	87.06

**Table 8:** Comparison of Normalized Life Time (Range 25 m).

Length of Pipeline (Meters)	Greedy Approach	ACO	GA	Proposed
500	0.6727	0.6946	0.6949	0.7067
1000	0.7000	0.7302	0.7377	0.7406
1500	0.7055	0.7453	0.7604	0.7805
2500	0.7248	0.7516	0.7782	0.7929
3500	0.7409	0.7781	0.7825	0.8084

**Table 9:** Comparison of Normalized Life Time (Range 50 m).

Length of Pipeline (Meters)	Greedy Approach	ACO	GA	Proposed
500	0.8105	0.8369	0.8372	0.8514
1000	0.8434	0.8798	0.8767	0.8923
1500	0.8500	0.8980	0.9161	0.9404
2500	0.9817	0.9417	0.9617	0.9890
3500	0.9167	0.9375	0.9428	0.9740

**Table 10:** Comparison of Normalized Life Time (Range 100 m).

Length of Pipeline (Meters)	Greedy Approach	ACO	GA	Proposed
500	0.8834	0.9122	0.9125	0.9280
1000	0.9193	0.9290	0.9296	0.9426
1500	0.9265	0.9328	0.9405	0.9512
2500	0.9315	0.9411	0.9513	0.9597
3500	0.9359	0.9469	0.9522	0.9667
